# Highly Selective Isotropic Etching of Si to SiGe Using CF_4_/O_2_/N_2_ Plasma for Advanced GAA Nanosheet Transistor

**DOI:** 10.3390/nano15191469

**Published:** 2025-09-25

**Authors:** Jiayang Li, Xin Sun, Ziqiang Huang, David Wei Zhang

**Affiliations:** 1 College of Integrated Circuits, Micro-Nano Electronics, Fudan University, Shanghai 200433, China; jiayangli21@m.fudan.edu.cn (J.L.); huangzq22@m.fudan.edu.cn (Z.H.); 2 School of Microelectronics, Fudan University, Shanghai 200433, China; 20112020027@fudan.edu.cn

**Keywords:** selective Si etching, isotropic etching, SiGe channel, gate all around, nanosheet transistor, remote plasma source

## Abstract

The paradigm shift from FinFET to gate-all-around nanosheet (GAA-NS) transistor architectures necessitates fundamental innovations in channel material engineering. This work addresses the critical challenge of pFET performance degradation in GAA-NS technologies through the development of an advanced selective etching process for strain-engineered SiGe channel formation. We present a systematic investigation of Si selective etching using CF_4_/O_2_/N_2_ gas mixture in a remote plasma source reactor. It is demonstrated that the addition of N_2_ to CF_4_/O_2_ plasmas significantly improves the selectivity of Si to SiGe (up to 58), by promoting NO* radical-induced passivation layer disruption on Si surfaces. Furthermore, an increase in the F:O ratio has been shown to mitigate stress-induced lateral micro-trenching (“Si-tip”), achieving near-zero tip length at high CF_4_ flow (500 sccm) while retaining selectivity (>40). Transmission electron microscopy and energy-dispersive X-ray spectroscopy confirm the complete removal of the Si sacrificial layer with minimal SiGe channel loss, validating the process for high-performance SiGe GAA-NS FET integration. These findings provide critical insights into strain-engineered SiGe channel fabrication, enabling balanced NFET/PFET performance in next-generation semiconductor technologies.

## 1. Introduction

The relentless pursuit of transistor miniaturization in the semiconductor industry has driven the evolution of device architectures from planar transistors to FinFETs and, more recently, to gate-all-around nanosheet field-effect transistors (GAA-NS FETs) [1,2,3,4]. The primary challenges limiting further scaling down of FinFETs are maintaining high drive current, minimizing off-state leakage, and controlling short-channel effects [5,6,7]. GAA-NS FETs, characterized by vertically stacked Si/SiGe nanosheets, offer superior gate controllability and design flexibility [6]. When the transistor architecture transitions from FinFETs to GAAFETs, the dominant conducting surface of the channel shifts from the (110) crystal plane to the (100) plane [8]. This change leads to a significant reduction in hole mobility, accompanied by a concurrent increase in electron mobility. The resulting asymmetry exacerbates the inherent imbalance between n-type (NFET) and p-type (PFET) transistor currents, presenting critical challenges for circuit performance and power efficiency in advanced technologies.

It is evident that the hole mobility of compression-strained SiGe channels is two to three times higher than that of unstrained Si channels [9,10,11,12]. This has resulted in the extensive utilization of SiGe channel in FinFET-based nodes (e.g., TSMC’s 5 nm to 3 nm process) to address the degradation of PFET performance [13,14,15,16]. However, the transition to GAA architectures disrupts the direct applicability of conventional SiGe channel integration methods. The GAA-NS FETs are fabricated through the heteroepitaxial growth of alternating Si and SiGe layers, which are patterned and vertically recessed to expose the layers laterally. A highly selective isotropic etching process between the sacrificial layers and the target channel layers becomes essential. Several studies have been published on the use of SiGe as sacrificial layers to be selectively etched, for the fabrication of Si GAA-NS FETs [17,18,19,20,21]. The development of the reverse process remains comparatively underexplored. The selective etching of SiGe to Si is primarily facilitated by leveraging differences in chemical reactivity under halogen-based plasma conditions [22,23,24,25,26,27,28]. For example, the lower activation energy required to break Ge-Ge and Si-Ge bonds compared to Si-Si bonds enables selective SiGe removal in fluorine-rich environments [23]. Conversely, the selective etching of Si to SiGe is achieved through differences in oxidation layer formation, or the preferential development of an etch-inhibiting passivation layer on SiGe [29,30,31,32]. Recent studies have demonstrated that in CF_4_/O_2_ plasma, Ge exhibits a higher affinity for oxygen (O) than both Si and F. Furthermore, it has been established that incorporating nitrogen (N_2_) into the CF_4_/O_2_ plasma significantly enhances the selectivity of the etching process. Current integration approaches of SiGe GAA-NS FETs face a critical roadblock: the inability to selectively etch Si relative to SiGe with sufficient precision to preserve the SiGe channel geometry (i.e., little or no thinning, no rounding at the entrance of nanosheets, and so on).

This work presents a systematic investigation of Si selective dry etching based on a CF_4_/O_2_/N_2_ gas mixture, targeting the integration of strain-engineered SiGe channels in GAA-NS FETs. The process was developed in a remote plasma source (RPS) reactor. We explore the interplay between gas composition (O_2_, N_2_, and O:F ratio), etching selectivity, and etching profile. By correlating plasma conditions with surface reaction dynamics, we demonstrate that optimized N_2_ addition enhances selectivity of Si to SiGe, while optimized F:O ratio mitigates stress-induced Si-tip. The influence of elevated F:O ratios on Si-tip length was systematically investigated by modulating the CF_4_/O_2_ flow rates within a CF_4_/O_2_/N_2_ gas mixture via two distinct approaches. At fixed CF_4_ and N_2_ flows, the Si-tip length decreases monotonically with reduced O_2_ flow, accompanied by a concomitant loss of etch selectivity. Conversely, at fixed O_2_ and N_2_ flows, raising the CF_4_ flow shortens the Si-tip length monotonically, approaching complete suppression at 500 sccm. These findings indicate that enhancing CF_4_ concentration in the CF_4_/O_2_/N_2_ gas mixture effectively inhibits Si-tip formation while maintaining high Si-to-SiGe etching selectivity. Our findings provide critical insights into achieving SiGe channel definition with minimal geometry change, paving the way for balanced NFET/PFET performance in next-generation GAA-NS technologies.

## 2. Materials and Methods

In this study, a commercially available multilayer stacked Si/SiGe superlattice wafer was used to characterize the Si selective etching process. The wafer consists of Si layers with a thickness of 30 nm, SiGe layers with a thickness of 50 nm, and a Ge content of 25% in the SiGe layers. The Si/SiGe superlattices were epitaxially grown on Si substrates using Reduced Pressure Chemical Vapor Deposition (RPCVD) equipment, ensuring that the SiGe layers share the same crystal structure and closely matched lattice constants as the Si substrate. As a result, the Si layer remains unstrained, while the SiGe layer is subjected to compressive strain. The Si selective etching process was carried out on a 200 mm etching platform consisting of two reactors connected via a vacuum transfer chamber: an inductively couple plasma (ICP) reactor for anisotropic etching of the hard mask and Si/SiGe superlattices, and an RPS reactor for isotropic and selective etching of the Si. In the RPS reactor, the plasma is generated by the application of a microwave discharge (2.45 GHz) in a quartz tube. A showerhead separates the substrate from the plasma, blocking charged species while allowing neutral particles to pass through the holes in the shower plate. Therefore, the substrate is exposed solely to reactive neutrals, allowing isotropic and chemical-type etching.

**Figure 1:**
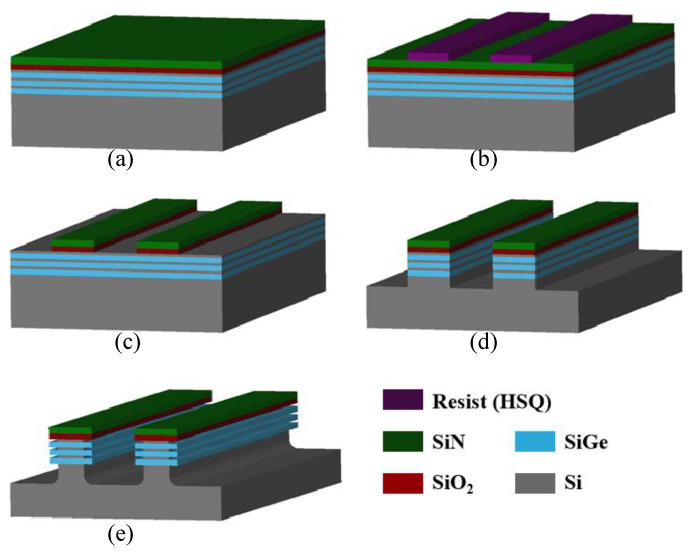
Process flow and test structure for evaluating Si-selective etching in Si/SiGe superlattices. (**a**) Si/SiGe superlattices epitaxy and hard mask deposition; (**b**) Pattern definition by EBL; (**c**) Hard mask etching by ICP; (**d**) Si/SiGe superlattices etching by ICP; (**e**) Si selective etching by RPS.

The test structure and process flow that were designed for the study of the Si selective etching process are shown in Figure 1. The pattern structures were fabricated as follows: First, a 60 nm-thick SiN layer and a 90 nm-thick SiO_2_ layer were sequentially deposited on Si/SiGe superlattice wafer using plasma enhanced chemical vapor deposition (PECVD) to form the hard mask (Figure 1a). Electron beam lithography (EBL) was then used to define the patterns, which were designed as rectangular arrays with a pitch of 1 μm, a length of 20 μm, and a width of 500 nm (Figure 1b). The E-beam resist used in this work is Dow Corning’s XR-1541 (Dow Corning Corporation, Midland, MI, USA), developed in a standard aqueous developer (0.26 N TMAH). After that, the patterns were transferred sequentially to the hard mask and Si/SiGe superlattices using anisotropic etching processes, allowing the buried Si and SiGe layers to be exposed to the etching species (Figure 1c,d). The etching of the SiO_2_ hard mask was performed using plasma generated from a 200 sccm CF_4_/200 sccm CHF_3_/20 sccm O_2_/600 sccm He gas mixture (source power 700 W, bias power 200 W, pressure 15 mTorr, temperature 50 °C), while the SiN hard mask was etched with plasma from a 200 sccm CH_3_F/100 sccm O_2_/150 sccm He gas mixture (source power 400 W, bias power 180 W, pressure 15 mTorr, temperature 50 °C). The etching of the Si/SiGe superlattices involved CF_4_ for the breakthrough step, followed by 280 sccm Cl_2_/120 sccm HBr/500 sccm He for the main etch (source power 360 W, bias power 380 W, pressure 10 mTorr, temperature 50 °C). The etching of the SiO_2_ hard mask was performed using plasma generated from a CF_4_/CHF_3_/O_2_/He gas mixture, while the SiN hard mask was etched with plasma from a CH_3_F/O_2_/He gas mixture. The etching of the Si/SiGe superlattices involved CF_4_ for the breakthrough step, followed by Cl_2_/HBr/He for the main etch. The anisotropic etching processes were performed in the ICP reactor. To remove remaining by-products and the oxidized layer on the pattern surface, O_2_ plasma-based dry etching (200 sccm O_2_, source power 600 W, bias power 0 W, pressure 10 mTorr, temperature 50 °C, process time 20 s) and wet cleaning (dip in 1% DHF for 60 s) were performed. Finally, the Si/SiGe superlattices, cut into 2 × 2 cm coupon wafers, were fixed on an 8-inch carrier wafer and transferred to the RPS reactor for Si selective etching (Figure 1e). In the RPS reactor, the wafer is placed on an electrostatic chuck, which regulate the requisite temperature for the etching. It is notable that a number of the process parameters were maintained across all studies. For instance, the microwave source power was set to 1000 W, the chamber pressure was regulated to 1.2 Torr, and the chuck temperature was maintained at 25 °C.

Leveraging the multilayer Si/SiGe stack as test structure, it is possible to observe and evaluate the selectivity and etching rate of the process by scanning electron microscopy (SEM, HITACHI SU8030, Hitachi, Tokyo Japan) and transmission electron microscopy (TEM, Thermo Fisher Talos-F200, Thermo Fisher Scientific, Waltham, MA, USA). The etch amounts were determined by measuring the difference in the remaining Si and SiGe before and after the Si selective etching process, which was then used to calculate the etching rate and selectivity. Furthermore, no deliberate tilt was applied during the process of SEM imaging.

## 3. Results

**Figure 2:**
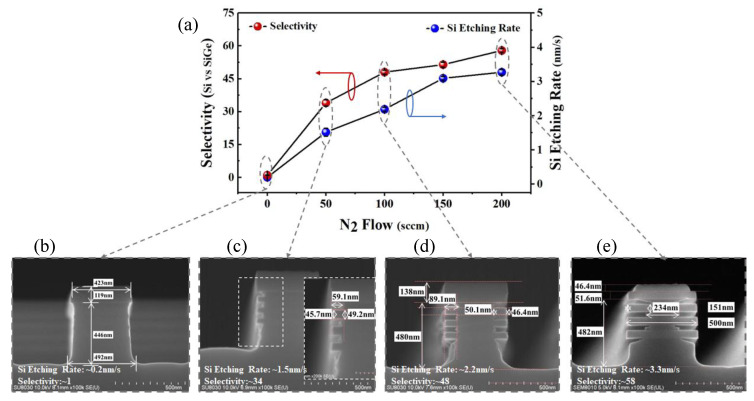
(**a**) Trends in the Si etching rate and selectivity for the Si selective etching process with different N_2_ flows (ranging from 0 to 200 sccm); Cross-sectional SEM images at (**b**) 0 sccm, (**c**) 50 sccm, (**d**) 100 sccm and (**e**) 200 sccm N_2_.

In order to have a better understanding of the role of N_2_ addition to the CF_4_/O_2_ plasma, we have studied the relationship between selectivity of Si to SiGe and the N_2_ concentration fed in the gas mixture. When the flows of CF_4_ and O_2_ were set at 300 sccm and 400 sccm, respectively, the trends in the Si etching rate and selectivity for the Si selective etching process with different N_2_ flows (ranging from 0 to 200 sccm) are shown in Figure 2a. Figure 2b–d presents the cross-sectional SEM images corresponding to the varying N_2_ flows. In the absence of N_2_, the selectivity of the etching process is approximately 1, indicating that the CF_4_/O_2_ gas mixture-based etching process exhibits no selectivity between Si and SiGe. Upon introducing N_2_ into the CF_4_/O_2_ plasma, the selectivity of the process increases significantly. Specifically, when the N_2_ flow is 50 sccm, the Si etching rate is approximately 1.5 nm/s, and the selectivity of Si to SiGe reaches about 34. As the N_2_ flow rate is increased to 100 sccm, the Si etching rate rises to approximately 2.2 nm/s, and the selectivity increases to around 48. At 150 sccm N_2_ flow, the Si etching rate further increases to approximately 3.1 nm/s, with the selectivity reaching about 51. Finally, when the N_2_ flow is 200 sccm, the Si etching rate increases slightly to 3.3 nm/s, while the selectivity rises to 58. The results demonstrate that, within a certain range, increasing the N_2_ concentration fed in the CF_4_/O_2_ gas mixture enhances Si etching rate and selectivity of the process.

**Figure 3:**
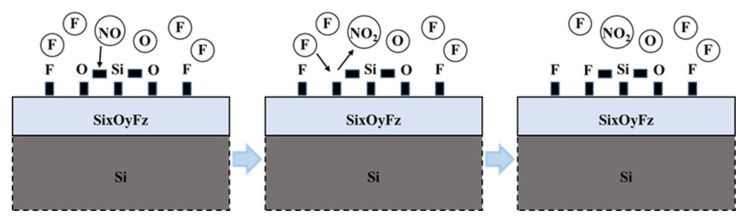
Proposed surface reaction mechanism: NO*-mediated disruption of SixOyFz passivation layer enabling F* radicals access to Si surface.

In the plasma based on CF_4_/O_2_, there are sufficient F* radicals and O* radicals. The F* radicals act as etchants and react with Si and SiGe of the superlattices to produce gaseous by-products SiF_4_ and GeF_4_ [24]. The O* radicals oxidize Si and SiGe, forming oxide layers SiOx and SiGeOx on the Si/SiGe superlattices surface [27]. The presence of oxide layers has been demonstrated to induce a passivation effect during the etching process. Furthermore, it has been demonstrated that when the by-products SiF_4_ and GeF_4_, which are generated during the process, are exposed to oxygen-rich plasma, a reaction layer is deposited on the surface of the pattern structure, leading to the cessation of the etching process [29]. The introduction of N_2_ into the CF_4_/O_2_ plasma altered the etchant species in the etching chamber. The newly introduced etchants, such as NO* radicals, have been shown to play a pivotal role in achieving high selectivity for Si to SiGe. The study by V. Caubet has suggested that NO* radicals can weaken the bond energy of Si-O bonds, facilitating their breakage and leading to the creation of new reactive sites on the Si surface [29]. It is reported that NO* have an energy of around 6 eV which is greater than the bonding energy of the oxide bonds. This mechanism allows F* radicals to combine more efficiently with Si atoms, thereby promoting the formation of volatile products and significantly accelerating the Si etching. The presence of O_2_ in the gas mixture leads to the generation of a significant amount of O* radicals within the etching chamber. It is important to note that the addition of N_2_ to CF_4_/O_2_ plasma enables a higher dissociation degree of O_2_ molecules, thereby resulting in a greater number of available O* radicals. These reactive O* radicals promote the formation of a reaction layer on the Si surface containing Si, O, and F elements (referred as a SixOyFz reaction layer). The reactive layer exerts a passivating effect, which can retard the etching of Si by F* radicals to a certain extent. However, upon the introduction of N_2_ into the CF_4_/O_2_ gas mixture, the generation of NO* radicals result in the rupture of the Si-O bond, leading to the formation of the gaseous byproduct NO_2_ and the creation of new reactive sites on the Si surface. These newly generated reactive sites are subsequently occupied by F* radicals, which then react with Si to form volatile SiF_4_ byproducts. The detailed reaction process of NO*-mediated disruption of the SixOyFz passivation layer, through which F* radicals are enabled to access the Si surface, is illustrated in Figure 3. This mechanism was originally proposed by V. Caubet et al. In summary, NO* radicals react with the SixOyFz reaction layer formed on the Si surface during the etching process, weakening or even eliminating the passivation effect of the reaction layer. It is well known that the bond energy of the Si-Ge bond (296 kJ/mol) is lower than that of the Si-Si bond (325 kJ/mol), making the Si-Ge bond more susceptible to breakage during the reaction [32]. Consequently, passivation layers (mixed layers of SixOyFz and GexOyFz) form on the SiGe surface earlier than on the Si surface. Moreover, the thicker passivation layer on the SiGe surface than on the Si surface was observed under the same conditions as reported by S. Rachidi et al. [32]. The SiGe etching rate is then greatly decreased compared to the Si etching rate, resulting in a high selectivity. However, it is unclear as to why the reaction layer formed on the SiGe surface would not interact with NO* radical in a similar manner and this has not yet been investigated to our knowledge. We hypothesize that the difference in composition between the passivation layers on Si and SiGe surfaces leads to their distinct reactions with NO* radicals—specifically, the passivation layer on the SiGe surface either does not react with NO* radicals or exhibits significantly weaker reaction intensity.

**Figure 4:**
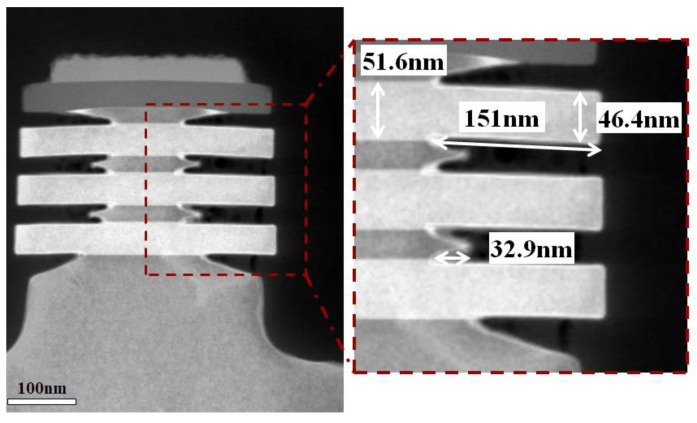
Cross-sectional TEM images of sample corresponding to the addition of 200 sccm N_2_ into the CF_4_/O_2_ (300/400 sccm) gas mixture.

As demonstrated in the cross-sectional SEM image in Figure 2, it is evident that following the Si selective etching process, the etch profile of Si layers in the Si/SiGe superlattices exhibits the formation of the lateral micro-trench (hereafter referred to as “Si-tip”). In order to further analyze the microstructural features of the Si-tip, the sample corresponding to the 200 sccm N_2_ flow was recharacterized using TEM, and the cross-sectional TEM image are shown in Figure 4. The Si-tips formed by the Si layers between the SiGe layers are bilaterally tapered, while the Si-tips formed by the top Si layer show single-sided tapered. The etch profile of the Si layer does not demonstrate consistency with the isotropic etching characteristic exhibited by the radical-based chemical etching process, thus indicating that the transverse etching rate of the Si layer in the stacking direction varies during the process. This variation is identified as the primary cause of the Si-tip formation.

In the Si/SiGe superlattice wafer, the lattice mismatch between Si and SiGe induces compressive stresses in the SiGe layers. After transferring the pattern to the Si/SiGe superlattices, the emergence of the free edges lead to a partial release of the compressive stress in the SiGe layers [33,34,35,36,37]. The stress release exerts a stress effect on the adjacent Si layers, transforming it from an unstressed state to one of tensile stress [35,37]. Since the tensile stress in the Si layer originates from the stress release of the adjacent SiGe layer, the regions of the Si layer closer to the SiGe layer experience greater stress. Conversely, the central region of the Si layer is subjected to the least stress in the stacking direction. Experimental results reveal a positive correlation between the stress and the Si etching rate: areas experiencing higher stress, such as those near the SiGe layer, exhibit a faster etching rate, while areas under lower stress, such as the central part of the Si layer, have a relatively slower etching rate. The portion of the top Si layer adjacent to the hard mask is relatively less influenced by the stress of the SiGe layer, resulting in a relatively small Si etching amount in this region of the Si/SiGe superlattices. The Si substrate, which remains largely unaffected by the stress of the SiGe layer, exhibits minimal etching amount. Therefore, we speculate that the Si etching rate is stress-dependent, with stress enhancing the Si etching rate during the selective etching process and contributing to the formation of the Si-tip. However, the effect of stress on the intrinsic mechanisms of the Si etching process remains unclear. Christopher et al. have demonstrated that the modulation of the length of the Si-tip was accomplished by means of varying the F:O ratio in the etching chamber, by adjusting the flow ratio of NF_3_ and O_2_ in the NF_3_/O_2_/N_2_ gas mixture [30]. Furthermore, it was observed that the length of the Si-tip exhibited a significant decreasing trend as the F:O ratio in the etching chamber increased.

**Figure 5:**
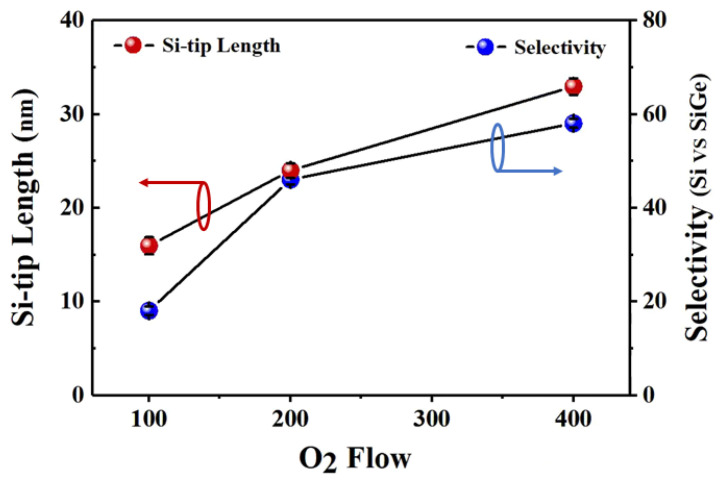
Si-tip length and selectivity as a function of the O_2_ flow variation in the CF_4_/N_2_ (300/200 sccm) gas mixture.

In this study, the effect of increasing the F:O ratio on the Si-tip length is investigated by adjusting the CF_4_ and O_2_ flow ratios in the CF_4_/O_2_/N_2_ gas mixture using two different approaches. In the first approach, the CF_4_ and N_2_ flows were kept constant while the O_2_ flow in the gas mixture was decreased. Figure 5 quantifies the O_2_ flow dependence of Si-tip formation and the selectivity of Si to SiGe. When the O_2_ flow was reduced from 400 sccm to 200 sccm, the Si-tip length decreased from 33 nm to 24 nm, accompanied by a significant decline in selectivity from 58 to 46. A further reduction in the O_2_ flow to 100 sccm resulted in a sharp deterioration in selectivity to below 20, while the Si-tip length remained approximately 16 nm. The etching mechanism in CF_4_/O_2_/N_2_ plasma involves a critical balance between etching and deposition processes. Experimental results demonstrate that reducing O_2_ flow decreases the concentration of reactive O* radicals, which subsequently: (1) enhances the direct interaction of F* radicals with Si surfaces, increasing the Si etching rate; and (2) reduces the oxidation of etching by-products, thereby diminishing passivation layer deposition. This shift toward etching-dominated reactions weakens stress-dependent etching effects, as evidenced by the progressive reduction in Si-tip length with decreasing O_2_ flow. The observed inverse correlation between O_2_ flow rate and Si etching rate (from 1.5 nm/s at 400 sccm to 3.3 nm/s at 200 sccm) confirms this mechanistic transition.

**Figure 6:**
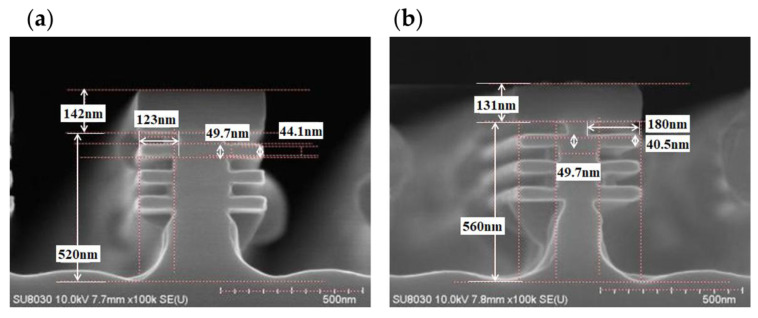
CF_4_ flow modulation effects: Cross-sectional SEM images of Si-tip elimination in Si/SiGe superlattices at (**a**) 400 sccm and (**b**) 500 sccm CF_4_ flow (O_2_/N_2_ fixed at 400/200 sccm). Figure 7 Channel release validation: (**a**) Cross-sectional TEM image of suspended SiGe nanosheets with complete Si removal, and corresponding EDS elemental maps of (**b**) Ge and (**c**) Si after the channel release.

In another approach, the O_2_ and N_2_ flows were kept constant while the CF_4_ flow in the gas mixture was increased. Figure 6 shows the cross-sectional SEM images corresponding to the CF_4_ flow of 400 sccm and 500 sccm. As the CF_4_ flow increases, the length of the Si-tip gradually decreases, and when the CF_4_ flow reaches 500 sccm, the length of the Si-tip is almost zero. It has been calculated that, when the CF_4_ flow is set at 400 sccm, the Si etching rate is approximately 4.1 nm/s, and the selectivity of Si to SiGe is approximately 44. Upon increasing the CF_4_ flow to 500 sccm, the Si etching rate rises significantly to 6.0 nm/s, while the selectivity exhibits a marginal decline to 39, which remains well within the acceptable range for practical applications. The results demonstrate that increasing the CF_4_ concentration in the CF_4_/O_2_/N_2_ gas mixture effectively suppresses Si-tip formation while preserving high etching selectivity of Si to SiGe. This phenomenon can be attributed to the enhanced generation of reactive F* radicals at higher CF_4_ flows, which intensifies the etching-dominated reaction mechanism. Concurrently, the constant O_2_ flow ensures that the deposition produced by the oxidation of etching reaction by-products is not significantly affected. Consequently, while Si-tip is eliminated, a high etching selectivity is retained. As a result, the optimized gas mixture achieves complete Si-tip elimination without compromising etching selectivity, making it a viable approach for precise SiGe nanosheets fabrication.

**Figure 7:**
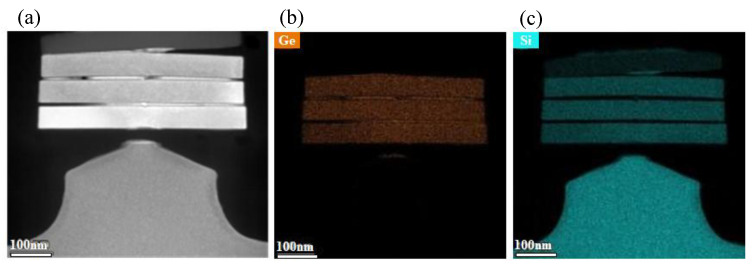
Channel release validation: (**a**) Cross-sectional TEM image of suspended SiGe nanosheets with complete Si removal, and corresponding EDS elemental maps of (**b**) Ge and (**c**) Si after the channel release.

In the fabrication of SiGe channel nanosheet FETs, the critical channel release step involves the complete removal of the Si sacrificial layer from Si/SiGe superlattices to form suspended stacked SiGe channels [17,25]. To minimize SiGe channel loss during the channel release process, the Si etching process must exhibit extremely high etch selectivity. Therefore, this work employs the process conditions with the highest etching selectivity for channel release, with the following parameters: reaction temperature maintained at 25 °C, chamber pressure at 1.2 Torr, source RF power at 1000 W, and gas flow of 300 sccm CF_4_, 400 sccm O_2_, and 200 sccm N_2_. Figure 7 presents the cross-sectional TEM image of the Si/SiGe stacked structure and the corresponding energy-dispersive X-ray spectroscopy (EDS) elemental maps of Ge and Si after the channel release. It can be observed that the Si sacrificial layer has been completely removed, while the SiGe channel layer has only suffered minimal etching loss. Notably, in our test structure—which intentionally omitted epitaxial source/drain regions for process evaluation—the released SiGe channels collapsed into a stacked configuration due to the absence of external support structures.

## 4. Conclusions

This study presents a comprehensive optimization of Si selective etching for SiGe channel integration in GAA-NS FETs, addressing key challenges in selectivity and profile control. The introduction of N_2_ into CF_4_/O_2_ plasma was found to enhance selectivity by up to 58, attributed to NO* radicals disrupting the Si surface passivation layer and accelerating Si etching. The presence of NO* radicals is pivotal in the process of selectivity, as they selectively rupture Si-O bonds. In contrast, SiGe remains protected by the passivation layer. Additionally, stress-induced Si-tip formation was effectively suppressed by tuning the F:O ratio, with higher CF_4_ flows (500 sccm) eliminating the Si-tip without compromising selectivity (>40). The optimized process (300 sccm CF_4_, 400 sccm O_2_, 200 sccm N_2_) enabled complete Si sacrificial layer removal with negligible SiGe thinning, as validated by TEM and EDS. This work establishes a robust dry-etching strategy for strain-engineered SiGe channels, paving the way for performance-scaled GAA-NS FETs with balanced NFET/PFET drive currents.

## Data Availability

The data presented in this study are available on request from the corresponding author.
